# Novel PGM3 mutation in two siblings with combined immunodeficiency and childhood bullous pemphigoid: a case report and review of the literature

**DOI:** 10.1186/s13223-022-00749-0

**Published:** 2022-12-24

**Authors:** Mazdak Fallahi, Mahnaz Jamee, Javad Enayat, Fahimeh Abdollahimajd, Mehrnaz Mesdaghi, Maliheh Khoddami, Anna Segarra-Roca, Alexandra Frohne, Jasmin Dmytrus, Mohammad Keramatipour, Mahboubeh Mansouri, Golnaz Eslamian, Shahrzad Fallah, Kaan Boztug, Zahra Chavoshzadeh

**Affiliations:** 1 Immunology and Allergy Department, Mofid Children’s Hospital, Shahid Beheshti University of Medical Sciences, Tehran, 15514-15468 Iran; 2 Pediatric Nephrology Research Center, Research Institute for Children’s Health, Shahid Beheshti University of Medical Sciences, Tehran, 15514-15468 Iran; 3 Skin Research Center, Shahid Beheshti University of Medical Sciences, Tehran, Iran; 4 Clinical Research Development Unit of Shohada-E Tajrish Hospital, Shahid Beheshti University of Medical Sciences, Tehran, Iran; 5 Pediatric Pathology Research Center, Research Institute for Children’s Health, Shahid Beheshti University of Medical Sciences, Tehran, Iran; 6 Ludwig Boltzmann Institute for Rare and Undiagnosed Diseases, Vienna, Austria; 7 St. Anna Children’s Cancer Research Institute (CCRI), Vienna, Austria; 8 Department of Medical Genetics, School of Medicine, Tehran University of Medical Sciences, Tehran, Iran; 9 CeMM Research Center for Molecular Medicine of the Austrian Academy of Sciences, Vienna, Austria; 10 Department of Pediatrics and Adolescent Medicine, Medical University of Vienna, Vienna, Austria; 11 St. Anna Children’s Hospital, Department of Pediatrics and Adolescent Medicine, Medical University of Vienna, Vienna, Austria

**Keywords:** PGM3 deficiency, Inborn errors of immunity, Skin, Blister, Case report

## Abstract

**Background:**

Bullous pemphigoid is the most common autoimmune subepidermal blistering disorder with a low incidence in childhood. Combined immunodeficiencies (CIDs) are a group of monogenic inborn errors of immunity (IEIs) characterized by T- and B-cell dysfunction leading to recurrent infections, lymphoproliferation, predisposition to malignancy, and autoimmunity. Here, we report two Afghan siblings with a diagnosis of CID and extremely rare manifestation of diffuse bullous pemphigoid skin lesions.

**Case presentation:**

The older sibling (patient 1) was a 32-month-old male with facial dysmorphism, protracted diarrhea, failure to thrive, recurrent oral candidiasis, recurrent otitis media with tympanic membrane perforation, who had been previously diagnosed with CID. While he was under treatment with intravenous immunoglobulin (IVIg), he developed extensive blistering lesions, which were diagnosed as childhood bullous pemphigoid. Methylprednisolone and azathioprine were added to the regimen, which resulted in a remarkable improvement of the skin lesions and also the feeding condition. However,2 weeks later, he was re-admitted to the intensive care unit (ICU) and eventually died due to fulminant sepsis. Later, his 12-month-old sister (patient 2) with similar facial dysmorphism and a history of developmental delay, food allergy, recurrent oral candidiasis, and respiratory tract infections also developed blistering skin lesions. She was under treatment for occasional eczematous lesions, and had been receiving IVIg for 3 months due to low levels of immunoglobulins. Further immunologic workup showed an underlying CID and thus treatment with IVIg continued, gradually improving her clinical condition. The genetic study of both siblings revealed a novel homozygous mutation in exon 7 of the *PGM3* gene, c.845 T > C (p.Val282Ala).

**Conclusions:**

Dermatologic disorders may be the presenting sign in patients with CID and mutated *PGM3*. This case report further extends the spectrum of skin manifestations that could be observed in PGM3 deficiency and emphasizes the importance of considering CIDs during the assessment of skin disorders, particularly if they are extensive, recurrent, refractory to treatment, and/or associated with other signs of IEIs.

## Background

Pemphigoid skin diseases are a group of rare autoimmune blistering disorders (AIBD) characterized by the development of autoantibodies that target hemidesmosome proteins involved in the maintenance of the dermo-epidermal integrity [1]. Bullous pemphigoid (BP) is the most prevalent AIBD [2] and classically presents as subepidermal tense blisters arising on a pruritic erythematous or unaffected background [3]. BP usually affects the elderly, and while pediatric cases are rare, they are more commonly associated with widespread lesions and mucosa involvement [4]. Combined immunodeficiencies (CIDs) are monogenic inborn errors of immunity (IEIs) presenting with T- and B-cell dysfunction [5]. Aside from infectious complications, CIDs may be accompanied by a vast spectrum of autoimmune conditions, mainly including endocrinopathies, cytopenias, and enteropathies [6].

Herein, we describe two CID siblings with autoimmune BP lesions who were found to have a novel missense variant in the Phosphoglucomutase 3 (*PGM3)* gene. This report provides new insights into genotype–phenotype correlation as bullous pemphigoid has never been reported in PGM3 deficiency.

We also aimed to review the existing evidence on pemphigoid skin diseases reported in IEI patients and to investigate PGM3 deficiency in patients with CID and severe CID (SCID) phenotypes.

## Method

Medical data of both patients were obtained by direct interview with parents and investigating in- and outpatient medical documents after receiving written informed consent from parents. Demographic data included age, sex, first presentation, age at disease onset and diagnosis, and outcome. The laboratory data included complete cell blood counts, lymphocyte subsets (by flowcytometry), lymphocyte functional assays, serum immunoglobulins (assessed using nephelometry and enzyme-linked immunosorbent assay), antibody titers to vaccinations, Nitroblue tetrazolium (NBT), and skin biopsy. Clinical diagnosis of CID has been established according to the European Society for Immunodeficiencies criteria [7].

Whole exome sequencing (WES) was performed on blood samples of both siblings in the Ludwig Boltzmann Institute for Rare and Undiagnosed Diseases, Vienna, Austria, using the Illumina Nextera DNA Flex Library Exome Kit for library preparation. An Illumina HiSeq3000/4000 instrument was used for 75-bp paired-end sequencing as previously described [8, 9]. Briefly, reads were aligned to the human genome version 19 by means of the Burrows-Wheeler Aligner (BWA). VEP was used for annotating single nucleotide variants (SNVs) and insertions/deletions lists. The obtained list was then filtered according to the presence of variants with a minor allele frequency (MAF) > 0.01 in 1000 Genomes, gnomAD, and dbSNP build 149. After further filtering steps for nonsense, missense, and splice-site variants using the DART software, an internal database was used to filter for recurrent variants. Moreover, variants were prioritized using tools, such as SIFT, Polyphen-2 and the combined annotation dependent depletion (CADD) score [10], that predict the deleteriousness of a present variant. The variant was confirmed through PCR amplification followed by Sanger sequencing in the Watson genetic laboratory, Tehran, Iran.

The literature search for reported IEI patients with pemphigoid blistering diseases was conducted in PubMed, Web of Science, and Scopus, applying the following keywords: “primary immunodeficiency”, “inborn error of immunity”, “congenital immunodeficiency syndromes”, “inherited immunodeficiency diseases”, in combination with subsequent terminology: “bullous pemphigoid”, “pemphigoid(s)”, “blisters”, and “bullous lesion”. Reference lists of all full-text articles and major reviews were manually searched for additional studies.

## Case presentations

Patient 1 was a 32-month-old male born at term to consanguineous Afghan parents living in Iran. At birth, he was noted to have facial dysmorphism with low set ears and hypotelorism but no skeletal abnormalities. The parents reported delay in the separation of umbilical cord by 40 days. He had received routine vaccination with no adverse effect. In his early months of life, he suffered from generalized eczematous lesions and recurrent episodes of oral candidiasis. At the age of 8 months, he developed two prominent erythematous skin lesions on the dorsal surfaces of the hands, which were found to be fungal infections through skin biopsy. He also suffered from protracted non-bloody diarrhea since the age of 14 months leading to hospitalizations. Food allergy, growth failure, and recurrent otitis media with tympanic membrane perforation were among other comorbidities.

When he was 16 months old, laboratory evaluation revealed lymphopenia, neutropenia, thrombocytosis, and increased levels of C-reactive protein (CRP) (Table 1). The serum levels of IgM and IgE were increased but IgG and IgA levels were in the normal range with respect to the age-matched reference values. Flow cytometry showed low CD4^+^ T cells and CD19^+^ B cells but normal natural killer (NK) cells. The specific antibody titers to diphtheria and tetanus were not protective. The Nitro blue tetrazolium (NBT) test was normal. The sputum smear and culture were negative for Bacillus Calmette-Guérin (BCG). The quantitative polymerase chain reaction (PCR) for human immunodeficiency viruses (HIV) was negative. According to the European Society for Immunodeficiency (ESID) criteria, the diagnosis of CID was established. He received extended-spectrum antibiotics and amphotericin B, and then prophylaxis with fluconazole and trimethoprim-sulfamethoxazole was initiated. The signs and symptoms were further treated with intravenous immunoglobulin (IVIg) substitution (500 mg/Kg/month) for about 1 year.

**Table 1:** Summary of Laboratory Investigations in Two Siblings at the Time of Diagnosis

Laboratory parameters	Patient 1 (age: 16 months)	Patient 2 (age: 12 months)	Reference value
WBC (cells/mm^3^)	**5700**	**3900**	6000–17000
Lymphocyte (cells/mm^3^)	**1938**	**663**	3000–9500
Neutrophil (cells/mm^3^)	**1026**	2496	1500–8500
Eosinophil (cells/mm^3^)	**114**	195	165–465
IgG (mg/dL)	849	875^†^	246–904
IgM (mg/dL)	**256**	44	40–143
IgA (mg/dL)	47	**174**	27–66
IgE (IU/mL)	**475**	**170**	Up to 68
Anti-D IgG (IU/mL)	** < 0.01**	**0.006**	< 0.1: No response 0.1–1: Poor response ˃1: Normal response
Anti-T IgG (IU/mL)	**0.05**	**0.39**	< 0.1: No response 0.1–1: Poor response ˃1: Normal response
Plt (× 10^3^ cells/mm^3^)	**1091**	**738**	150–450
CD3 + T cells (% of lymphocytes)	79%	**30%**	50–90
CD4 + T cells (% of lymphocytes)	**12%**	22%	20–65
CD8 + T cells (% of lymphocytes)	50%	**2%**	5–40
CD19 + B cells (% of lymphocytes)	**2%**	27.5%	3–40
CD16 + NK cells (% of lymphocytes)	15%	16.7%	3–15
CD56 + NK cells (% of lymphocytes)	19%	21%	3–15
NBT	100%	100%	> 95
CRP	112	3	< 10
LTT			
PHA	4.8	4.9	≥ 3
BCG	**1.1**	1.4	≥ 2.5
Candida	**1.8**	2.0	≥ 2.5

Bold items indicate abnormal parameters according to the reference ranges for ages

*WBC* white blood cell, *Ig* immunoglobulin, *Anti-D* anti-diphtheria, *Anti-T* anti-tetanus, *Plt* platelet, *NK* natural killer, *CRP* C-reactive protein, *LTT* lymphocyte transformation test, *PHA* phytohemagglutinin, *BCG* Bacillus Calmette-Guérin

^†^While on exogenous immunoglobulin

**Fig. 1:**
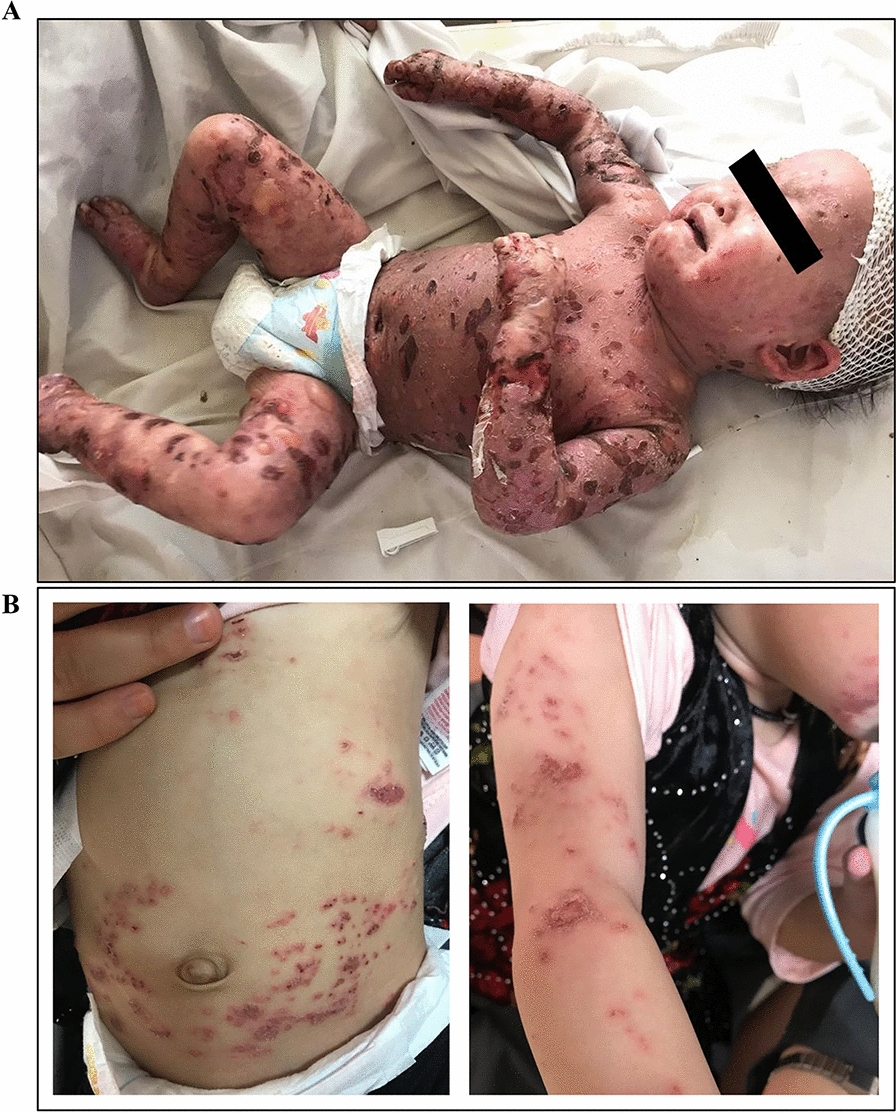
Diffuse bullous lesions in patient 1 **A** and 2 **B**

**Fig. 2:**
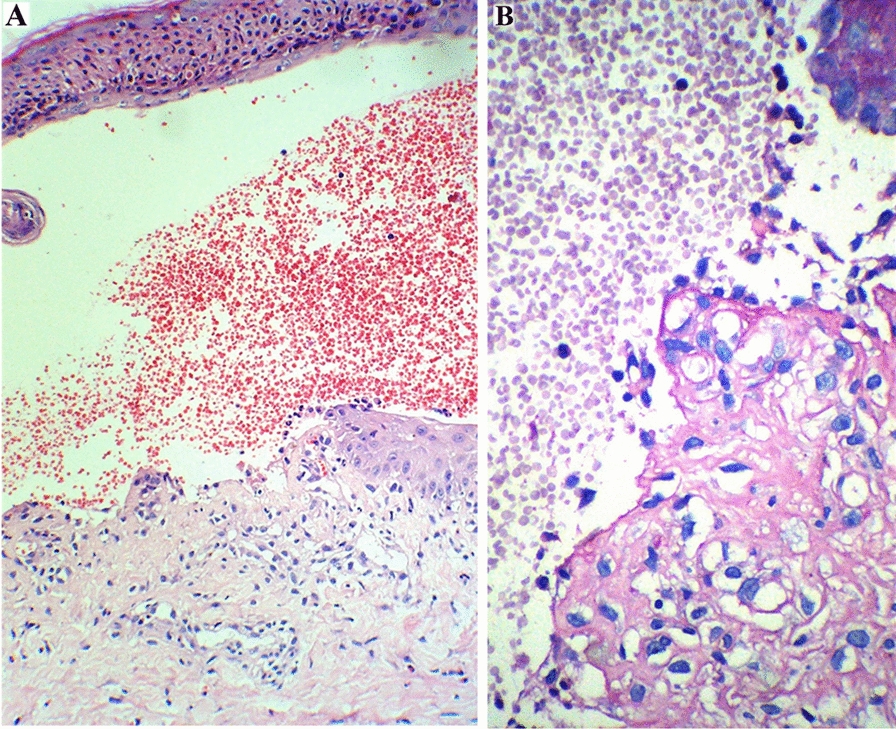
The histopathologic findings of skin lesions. **a** Subepidermal blister which appears intraepidermal located at the periphery as a result of epithelial regeneration. The blister is filled with many red blood cells and small number of neutrophils, eosinophils and lymphocytes, H&E × 200. **b** Periodic acid Schiff (PAS) positive basement membrane is focally presented at the base of the subepidermal blister, PAS × 400

He was referred to our hospital with a 6-month history of progressive bullous lesions. The lesions involved the face, trunk, palms, and soles, although the mucous membrane was intact (Fig. 1A). The bullae were mostly tense and a few had a thin roof and easily ruptured within 24 h. In the histopathologic examination, spongiotic epidermal reaction and subepidermal blisters associated with perivascular and interstitial infiltration of eosinophils and smaller numbers of neutrophils and lymphocytes were observed (Fig. 2). The immunohistochemistry study showed positive results for the CD1a, S100, and C kit proteins. Using direct immunofluorescence (DIF), linear deposition of IgG and C3 along the dermo-epidermal junction was found, compatible with childhood BP. He also developed hair loss and koilonychia. Methylprednisolone and azathioprine were added to the regimen, which resulted in a remarkable improvement of the skin lesions after 3 weeks. However, 2 weeks later, he was re-admitted to the intensive care unit (ICU) and eventually died due to fulminant sepsis.

One week after he passed away, patient 2 (his sister) presented at 1-year-old with severe eczematous skin lesions, which progressed to the forms of papulopustular and bullous rashes on the entire trunk and limbs and suffered from lesions similar to her sibling’s (Fig. 1B). She had facial dysmorphism comparable to her brother’s, had long been under treatment for occasional eczematous lesions, and had been receiving IVIg for 3 months due to low levels of immunoglobulins. No post-vaccination complication was reported. She also suffered from delayed umbilical cord separation (by 30 days), developmental delay, allergy to cow’s milk, recurrent oral candidiasis, and episodes of respiratory tract infections since infancy. Eventually, based on the basic immunologic workup, an underlying CID was suspected and treatment with IVIg continued. She is now well and in a relatively stable health condition. Later, the genetic study on blood samples of both siblings by WES revealed a novel homozygous ENST00000506587.5:c.845 T > C, p.Val282Ala variant in exon 7 of the *PGM3* gene [SIFT: deleterious, PolyPhen: probably damaging, CADD score: 25].

**Fig. 3:**
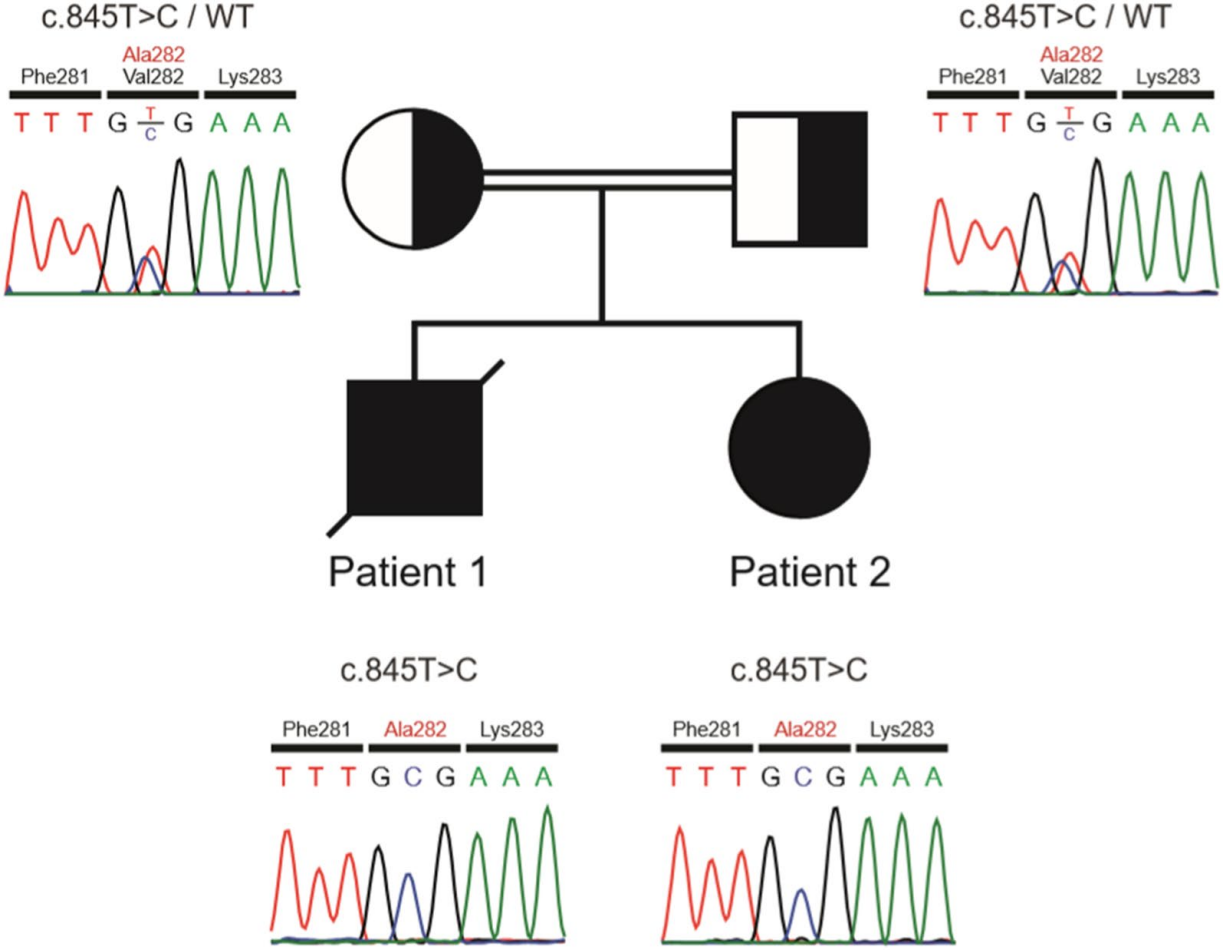
The family pedigree and chromatograms. Sanger sequencing confirmed a homozygous missense variant (c.845 T > C, p.Val282Ala) in exon 7 of the *PGM3* gene in the index patient **A**. The mother **B** and father **C** were heterozygous for the variant. *Squares, male; circle, female; solid symbols, affected subjects; slashed symbol, deceased subject*

Both parents were shown to be heterozygous for the variant (Fig. 3).

## Discussion

Cutaneous lesions may be the first or predominant presentation in patients with IEIs. The most commonly reported skin disorders include eczema, mucocutaneous candidiasis, skin abscess, granulomas, erythroderma, warts, molluscum contagiosum, alopecia, and vitiligo (for a detailed review, refer to [11]). Among others, pemphigoid skin diseases are extremely rare dermatologic manifestations in IEIs and there have been few reports of only 14 patients with these complications.

Pemphigoid skin diseases are autoimmune subepidermal bullous disorders encompassing different subtypes, namely BP, mucous membrane pemphigoid, epidermolysis bullosa acquisita, gestational pemphigoid, and anti-p200 pemphigoid, with BP being the most prevalent [1]. Although rare, the diagnosis of childhood BP is considered in young patients (≤ 18 years old) with tense subepidermal bullae and dermal infiltration, predominantly by eosinophils. Nonetheless, the definite diagnosis is ascertained by DIF showing linear deposition of IgG and/or C3 alongside the basement membrane zone or by the detection of circulating IgG autoantibodies against the basement membrane through indirect immunofluorescence [12].

The first report documenting childhood pemphigoid in a setting of immunodeficiency was made about two decades ago when Bloomfield et al. [13] reported an 8-month-old girl with thymic hypoplasia, autoimmune hemolytic anemia, T cell lymphopenia, and juvenile pemphigoid. She failed to respond to steroids and, despite adding sulphapyridine to the regimen, the bullae became extensive and she eventually died due to pulmonary edema. After that, clinically similar patients with immune dysregulation, polyendocrinopathy, enteropathy, X-linked (IPEX) [14–16] and IPEX-like syndrome [17, 18], hyper IgE syndrome (HIES) [19, 20], zeta-chain-associated protein kinase 70 (ZAP70) deficiency [21], and common variable immunodeficiency (CVID) [22] were reported to suffer from pemphigoid blistering conditions, including BP, pemphigoid nodularis, and mucous membrane pemphigoid.

A summary of the demographic and clinical features of IEI patients who had pemphigoid blistering disorder complications are provided in Table 2. It is worth noting that the focus of this review is on BP disorders and associated variants. There have also been reports of other similar childhood blistering disorders in IEI patients such as chronic granulomatous disease (CGD) with linear IgA dermatosis [23], auto-inflammation and phospholipase Cγ2 (PLCγ2)-associated antibody deficiency and immune dysregulation (APLAID) syndrome with early-onset blistering lesions [24], and SERPING1 mutation with bullous lesions at the site of angioedema [25] that are not discussed here.

**Table 2:** A summary of the demographic and clinical features of IEI patients with pemphigoid blistering diseases

Patient	Type of PIDs (Mutated gene)	Type of blistering disorder	Age (years)	Sex	Parental consanguinity	Origin	The onset of skin disorder (years)	Blister involvement	DIF	Other comorbidities	Treatment	D/A	References
1	CID (*PGM3*)	Bullous pemphigoid	2.7	M	Yes	Afghanistan	2.2	Face, trunk, extremities	IgG, C3	FTT, enteropathy, allergy, recurrent otitis media with tympanic membrane perforation, recurrent oral candidiasis, hair loss, koilonychia	Prednisolone, azathioprine	D	This report, 2022
2	CID (*PGM3*)	Bullous pemphigoid	1.0	F	Yes	Afghanistan	NA	Face, trunk, extremities	IgG, C3	Food allergy, eczema, FTT, Recurrent oral candidiasis, RTI, recurrent oral candidiasis	IVIg	A	This report, 2022
3	IPEX (*FOXP3*)	Bullous pemphigoid	7.0	M	No	Denmark	2.0	Face, extremities, gluteal region	IgG	FTT, Lymphoid interstitial pneumonia	Prednisolone, Azathioprine, MMF, CsA	A	Anderson et al. 2021
4	IPEX (*FOXP3*)	Pemphigoid nodularis	23.0	NA	NA	USA	NA	NA	NA	Recurrent RTI, Enteropathy, Eczema/psoriasis, Thyroiditis, Nephropathy, Hypogammaglobulinemia	Immunosuppressive, IVIg, HSCT	NA	Rosenberg et al. 2018 [13]
5	CVID (NA)	Mucous membrane pemphigoid	73.0	F	NA	USA	72.2	Oral mucosa	IgG, C3	No	Topical dexamethasone	A	Doll et al., 2017 [21]
6	ZAP70 Deficiency (*ZAP70*)	Bullous pemphigoid	2.0	M	No	USA	1.6	Face, trunk, extremities, oral mucosa	IgG	Inflammatory colitis, Hemophilia, Nephrotic Syndrome	HSCT	A	Chan et al., 2016 [20]
7	ZAP70 Deficiency (*ZAP70*)	Bullous pemphigoid	0.2	F	No	USA	0.1	Face, trunk, extremities	IgG	Growth failure, Inflammatory colitis, Proteinuria, autoimmune thyroiditis	HSCT	A	Chan et al., 2016 [20]
8	HIES (*STAT3*)	Bullous pemphigoid	30.0	F	NA	Indonesia	4.0	Entire body	NA	Esophagus stricture, Recurrent RTI, Lung TB, Alopecia, Hair loss, Anonychia, Anemia, Malnutrition	Prednisolone, Topical antibiotics	A	Budiyani et al. 2016 [18]
9	IPEX-like syndrome (*CD25/IL2RA*)	Bullous pemphigoid	8.0	F	Yes	Italy	1.0	NA	NA	Autoimmune enteropathy, CMV infection, Diffuse eczema, Autoimmune thyroiditis, Alopecia universalis, Lymphadenopathies	Plasmapheresis	A	Goudy et al. 2013 [16]
10	IPEX-like syndrome (NA)	Bullous pemphigoid	7.0	NA	NA	Italy	NA	NA	NA	Arthritis, AIHA, Autoimmune hepatitis, Enteropathy	Steroids, Rituximab, MMF, cyclophosphamide	NA	Barzaghi et al. 2012 [17]
11	HIES (NA)	Bullous pemphigoid	0.5	M	No	Turkey	0.2	Face, trunk, extremities, oral mucosa	IgG	Recurrent RTI, Recurrent oral thrush, Otitis, Severe eczema	Prednisolone, Antibiotics	A	Erbagci et al. 2008 [19]
12	CVID	IgA mucous membrane pemphigoid	46.0	F	NA	Canada	45.3	Conjunctiva	IgA	Eye disorder, meningioma, atrial myxoma	Dapsone, IVIG		Suwattee et al. 2004
13	IPEX (*FOXP3*)	Bullous pemphigoid, pemphigoid nodularis	14.0	M	No	USA	0.1	Face, trunk, extremities	IgG, C3	Autoimmune enteropathy, Recurrent RTI, Abscess, Asthma, growth failure, VZV and EBV infection	Prednisolone, CsA, Dapsone, Azathioprine, MTX, Hydroxyzine, SSRI, Topical tacrolimus, IVIg, Antibiotics, Rituximab	A	Ferguson et al. 2000 [14], McGinnes et al. 2006 [15]
14	NA	Juvenile pemphigoid	0.8	F	NA	UK	0.7	Face, trunk, extremities	IgG	Thymic hypoplasia, AIHA, T lymphopenia	Prednisolone, sulphapyridine	D	Bloomfield et al. 1982 [12]

*A* alive, *AIHA* autoimmune hemolytic anemia, *CGD* chronic granulomatous disease, *CID* combined immunodeficiency, *CMV* cytomegalovirus, *CsA* cyclosporine A, *CVID* common variable immunodeficiency, *D* dead, *DIF* direct immunofluorescence, *EBV* epstein-barr virus, *F* female, *FOXP3* forkhead box protein P3, *HIES* hyper IgE syndrome, *HSCT* hematopoietic stem cell transplantation, *Ig* immunoglobulin, *IVIg* intravenous immunoglobulin, *IL2RA* interleukin-2 receptor alpha chain, *IPEX* immune dysregulation, polyendocrinopathy, enteropathy, X-linked, *M* male, *MMF Mycophenolate* mofetil, *MTX* methotrexate, *NA* not available, *PID* primary immunodeficiency disorder, *RTI* respiratory tract infection, *STAT* signal transducer and activator of transcription, *TB* tuberculosis, *VZV* Varicella zoster virus, *ZAP70* zeta-chain-associated protein kinase 70

Intriguingly, five out of 14 reported IEI patients with BP disorders were diagnosed with IPEX or IPEX-like syndrome. This association may result from a low regulatory T cell (T reg) count [26], which is reported in 68% and 50% of patients with IPEX and IPEX-like syndrome, respectively [27]. In fact, recent studies have demonstrated that T reg cells play an important role in preventing the production of autoantibodies against BP180 and BP230 in human and mice models [28].

The protein encoded by *PGM3* is required for the reversible conversion of N-acetylglucosamine-6-phosphate (GlcNAc-6-P) to N-acetylglucosamine-1-phosphate (GlcNAc-1-P) during the synthesis of UDP-GlcNAc, with an intra- and extracellular structural role, as well as a role in cell signaling [29]. Variants in *PGM3* were first assumed to be responsible for autosomal recessive forms of hyper IgE syndrome, patients with CID/SCID phenotype and mutated *PGM3* are reported in the literature [30–33], mainly manifesting as facial dysmorphisms, skeletal abnormalities, neurologic disorders, renal disorders, and gastrointestinal complications, and less frequently congenital heart disorders, and recurrent respiratory tract infections. Most patients suffered from skin disorders mainly in the form of eczema or as a consequence of infections but none of them had BP as the patients depicted here. Therefore, underlying *PGM3* mutation should be suspected in (S)CID patients with facial and skeletal abnormalities.

To our knowledge, this is the first report of childhood bullous pemphigoid in the setting of PGM3 deficiency. Dermatologic disorders may be the presenting sign in patients with CID. In a cohort of 696 CID patients, about 11% of non-syndromic CID patients primarily presented with generalized eczema, skin infections, and abscesses, and almost half of the patients with syndromic CID developed dermatologic abnormalities during the course of the disease [34]. These findings emphasize the importance of considering CIDs during the assessment of skin disorders, particularly if they are extensive, recurrent, refractory to treatment, and/or associated with other signs of IEIs [11].

## Data Availability

The detailed laboratory data of two patients are available at supplementary material.

## Abbreviations

AIBD: Autoimmune blistering disorders
APLAID: Auto-inflammation and phospholipase Cγ2-associated antibody deficiency and immune dysregulation
BP: Bullous pemphigoid
CGD: Chronic granulomatous disease
CIDs: Combined immunodeficiencies
CRP: C-reactive protein
CVID: Common variable immunodeficiency
DIF: Direct immunofluorescence
HIES: Hyper IgE syndrome
HIV: Human immunodeficiency viruses
ICU: Intensive care unit
IEIs: Inborn errors of immunity
IPEX: Immune dysregulation, polyendocrinopathy, enteropathy, X-linked
IVIg: Intravenous immunoglobulin
MAF: Minor allele frequency
NBT: Nitro blue tetrazolium
NK: Natural killer
PCR: Polymerase chain reaction
PGM3: Phosphoglucomutase 3
SNVs: Single nucleotide variants
WES: Whole exome sequencing
ZAP70: Zeta-chain-associated protein kinase 70
